# Characterisation of extreme load responses of a 10-MW floating semi-submersible type wind turbine

**DOI:** 10.1016/j.heliyon.2023.e13728

**Published:** 2023-02-14

**Authors:** Yihan Xing, Shuaishuai Wang, Anuraj Karuvathil, Rajiv Balakrishna, Oleg Gaidai

**Affiliations:** a Department of Mechanical and Structural Engineering and Materials Science, University of Stavanger, Norway; b Norwegian University of Science and Technology, Trondheim, Norway; c Shanghai Engineering Research Centre of Marine Renewable Energy, College of Engineering Science and Technology, Shanghai Ocean University, Shanghai, China

**Keywords:** Floating offshore wind turbine, FAST, Extreme value analysis, ACER method, Gumbel method

## Abstract

The global average size of offshore wind turbines has increased steadily from 1.5 MW to 6 MW from 2000 to 2020. With this backdrop, the research community has recently looked at huge 10–15 MW class floating offshore wind turbines (FOWTs). The larger rotor, nacelle structure and tower have more significant structural flexibility. The larger structural flexibility, controller dynamics, aerodynamics, hydrodynamics, and various environmental conditions result in complex structural responses. The structural load effects of a very large FOWT could be more severe than that of the lower MW classes. Accurate quantification of the extreme dynamic responses of FOWT systems is essential in the design of the Ultimate Limit State (ULS) due to the fully-coupled interaction between the FOWT system and environmental conditions. Motivated by this, extreme responses of the 10 MW semi-submersible type FOWT are investigated using the average conditional exceedance rate (ACER) and Gumbel methods. Three operating conditions representing below-rated (U = 8 m/s), rated (U = 12 m/s) and above-rated (U = 16 m/s) regions were considered. The aim is to guide future research on large FOWTs by indicating the expected ULS loads.

## 1 Introduction

Offshore wind has been developing quickly over the past decade. In the last decade, there has been on average an annual growth of 22% in cumulative offshore wind power installed [1]. In 2020, the cumulative installed wind energy was 35 GW. This was 14 times higher than a decade ago. Further, it is estimated that there will be over 235 GW of new installations over the next decade, which demonstrates a great prospect.

One observation over the years in the technological development of wind turbines is that wind turbine capacities have consistently increased. This is particularly true of offshore wind turbines (OWTs). Larger wind turbine sizes enable the same power output with fewer turbines, foundations, converters, and cables and lower maintenance costs, thus reducing the overall cost of energy. The global average size of offshore wind turbines has increased steadily from 1.5 MW to 6 MW from 2000 to 2020 [1]. This trend continues with the research community recently starting conceptual studies for 15-20 MW-class offshore turbines, such as the IEA 15-MW offshore reference wind turbine [2].

Accurate quantification of the extreme dynamic responses of OWT systems is essential in the Ultimate Limit State (ULS) based design. Due to the fully-coupled interaction between the OWT system and environmental conditions, the responses are strongly nonlinear and highly dynamic. A robust set of design requirements must ensure that the extreme load effects over the entire design lifetime are correctly assessed with corresponding structural capacities designed in the OWT. Estimating these extreme load effects can be challenging. Direct calculation of extreme structural responses could obtain accurate results, but this method needs many dynamic simulations and substantial computational costs. As proposed in IEC 61400-3 [3], a statistical extrapolation method for ultimate strength analysis makes it possible to evaluate the extreme load effects of OWTs by using a much smaller amount of data, thereby saving a great deal of computational time.

Many studies have evaluated the effectiveness of various statistical extrapolation methods when used for the extreme load and load effect analysis for OWTs. Saha et al. [4] studied the extreme short-term responses of a jacket foundation of a 5 MW OWT. The authors studied the sensitivity of the extreme responses to sample sizes. Three extreme value analysis methods were investigated: (i) the mean up-crossing rate, (ii) the Weibull tail, and (iii) the global maxima. The up-crossing rate method was found to have better performance compared to the other two methods for Gaussian/non-Gaussian responses. Dimitrov [5] investigated the extraction of independent response peaks of wind turbine loads by comparing different extreme value methods applied to the land-based 10 MW DTU WT. The results showed that, under normal operating conditions, the statistical distribution of extreme loads could be reasonably estimated by the statistical load extrapolation method. In contrast, there are more uncertainties for transient load cases such as fault, storms, grip drops and emergency stops. Using measurement data, Lott and Cheng [6] presented different methods to perform statistical extrapolations of extreme loads at the blade root and tower base locations. The wind turbine studied was the Alpha Ventus wind turbine, a 5 MW bottom-fixed tripod-type OWT. It was shown that correctly selecting the fitting method, the distribution function, and the database is essential in determining the extrapolated extreme loads. A statistical extrapolation-based, stochastic programming formulation was presented by Cao et al. [7] to reduce long-term extreme loads in WTs. They used land-based 5 and 6-MW wind turbines where the power performance significantly improved without exceeding the mechanical capacities of the WTs. The extreme and fatigue load effects of 5 MW horizontal and vertical axis semi-submersible type FOWTs were compared by Cheng et al. [8]. The extreme responses of a 5 MW spar-type FOWT integrated with a torus wave and tidal energy convertors were studied by Li et al. [9] using an extrapolated up-crossing rate method. The tidal current convertors are mounted below the water a the left and right of the system. The wave nonlinearity effects on the fatigue and extreme responses of a 5 MW semi-submersible FOWT were investigated by Xu et al. [10].

Most studies on extreme structural response were performed on small-scale or medium-scale OWTs. Minimal effort has been devoted to very large (10–15 MW) floating OWTs. Very large FOWT has longer blades, larger swept areas, and taller tower heights, leading to larger aerodynamic loads. In addition, it has a heavier rotor-nacelle-assembly (RNA) system and a larger support structure which leads to larger inertial loads. Further, studies performed by Wang et al. [11,12] indicated that larger wind turbines could be at risk of resonance. The larger rotor, nacelle structure and tower could be sufficiently flexible so their natural frequencies can be close to the low-frequency wind and wave excitations. The generally more significant load effects mean that the structural load effects of a very large FOWT could be more severe than that of the lower MW classes. This highlights the importance of accurately quantifying the dynamic load effects of these very large FOWTs, which is paramount in their ULS design.

Motivated by the above, the present work will characterise the extreme structural responses of a 10-MW semi-submersible FOWT using the widely used Gumbel fitting and ACER methods. The paper will investigate the blades-hub, rotor-main shaft, and tower base-floating platform interfaces, which are considered critical locations in the FOWT. Representative operating conditions at below-rated (U = 8 m/s), rated (U = 12 m/s), and above-rated (U = 16 m/s) wind speeds are studied. The objective is to provide guidance on future research on very large OWTs by indicating the expected ULS loads.

## 2 System description

**Fig. 1:**
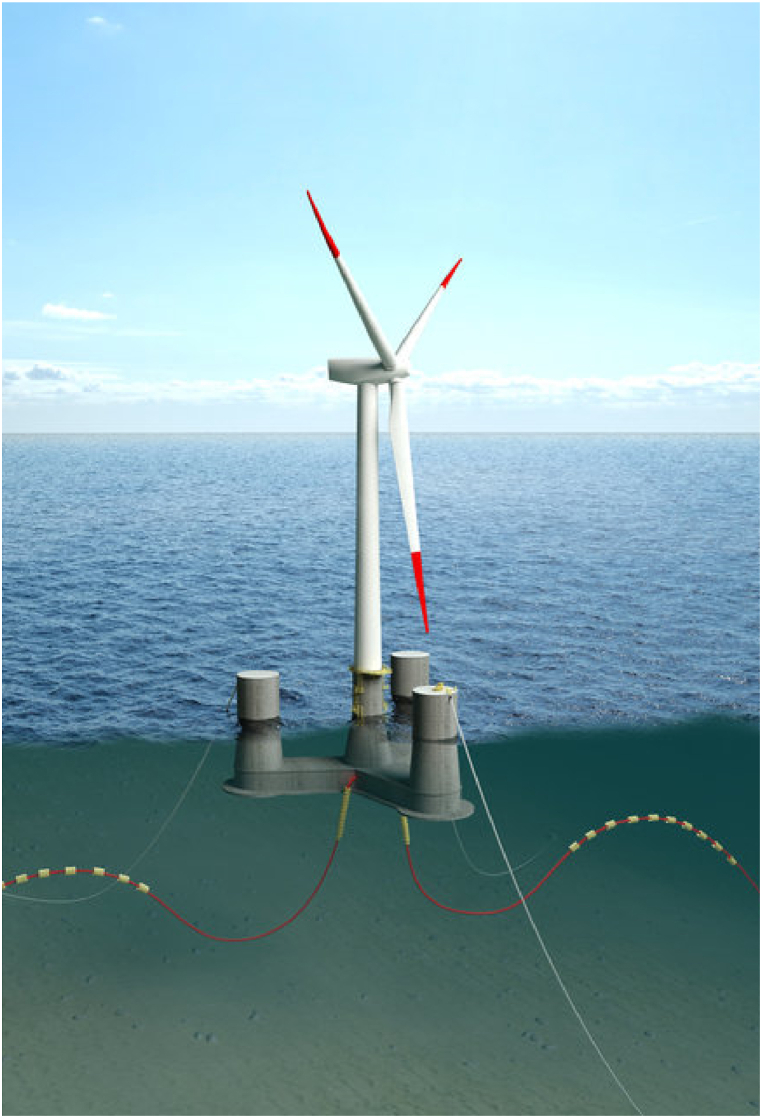
The 10-MW OO-star FOWT [13].

**Fig. 2:**
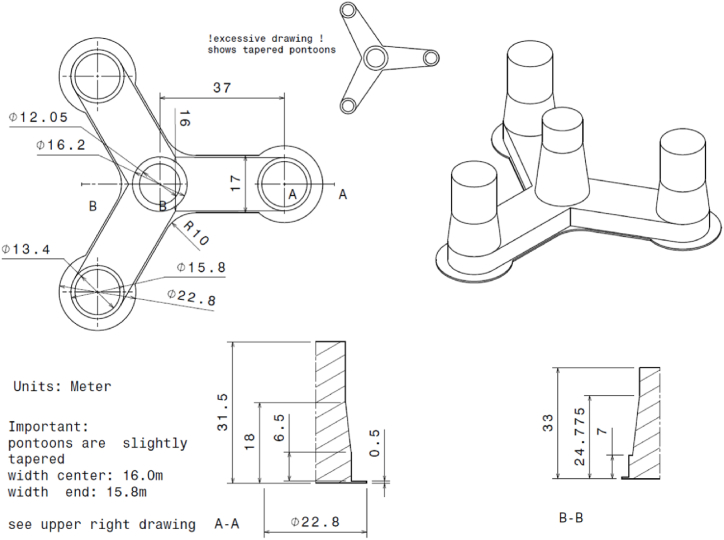
OO-Star floater's global dimensions [19].

The 10-MW OO-Star FOWT [13] studied is illustrated in Fig. 1 and presented in some detail in the following sub-sections (see Fig. 2).

### 2.1 DTU 10-MW reference wind turbine

The OO-Star FWT uses the DTU 10-MW reference wind turbine (RWT) [14] developed and studied by many researchers, such as Muggiasca et al. [15], Yu et al. [16], Wang et al. [17], and Hu et al. [18]. This is an International Electrotechnical Commission (IEC) Class 1A upwind wind turbine with three blades turning in a clockwise direction. The wind turbine uses collective blade pitch and variable generator speed controls. The key parameters are presented in Table 1.

**Table 1:** DTU 10-MW RWT's key parameters [14].

Parameter	Value
Rating	10-MW
Type	Upwind/3 blades
Control	Variable speed, collective pitch
Drivetrain	Medium-speed, multiple stage gearbox
Cut-in, rated and cut-out wind speed (m/s)	4, 11.4, 25
Minimum and maximum rotor speed (rpm)	6.0, 9.6
Maximum generator speed (rpm)	480
Rotor diameter (m)	178.3
Hub height (m)	119.0
Rotor mass (kg)	227,962
Nacelle mass (kg)	446,036
Tower mass (kg)	1.257 × 10^6^

### 2.2 OO-star floating sub-structure and mooring system

**Fig. 3:**
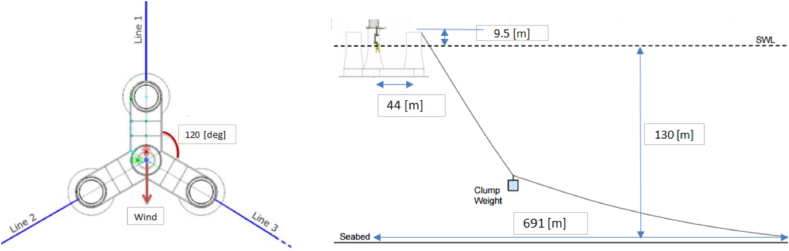
Sketch of the mooring system in the 10-MW FOWT (left: top view; right: side view) [19].

Dr.techn. Olav Olsen AS [13,14] developed the post-tensioned concrete-made, floating semi-submersible type sub-structure in the LIFES 50+ project [13]. The floater has three outer columns located radially and symmetrically to a central main column. These columns are connected to each other via rectangular stabs giving a star shape when viewed from the top. The station keeping is provided by three catenary mooring chains with additional clumped mass installed at the mid-length for increased tension, as shown in Fig. 3. The floater is illustrated in Fig. 2. Table 2 and Table 3 present the key parameters of the floater and the mooring system, respectively.

**Table 2:** The main properties for the 10-MW OO-Star wind floater.

Parameter	Value
Water depth (m)	130
Draft (m)	22
Tower-base interface above mean sea level (m)	11
Displacement (kg)	24,158
Overall gravity, including ballast (kg)	21,709
Roll and pitch inertia about center of gravity (kg∙m^2^)	1.4462 × 10^10^
Yaw inertia about center of gravity (kg∙m^2^)	1.63 × 10^10^
Center of gravity height below mean sea level (m)	15.23
Center of buoyancy height below mean sea level (m)	14.236

**Table 3:** The main properties for the mooring system of the 10-MW FOWT.

Parameter	Value
Radius to anchors from platform centerline (m)	691
Anchor position below MSL (m)	130
Initial vertical position of clump mass below MSL (m)	90.45
Initial radius to clump mass from centerline (m)	148.6
Length of clump mass upper segment (m)	118
Length of clump mass lower segment (m)	585
Equivalent weight per length in water (N/m)	3200.6
Extentional stiffness (N/m)	1.506 × 10^9^

## 3 Methodology

### 3.1 Aero-hydro-elastic-servo dynamic analysis of the 10-MW FOWT

The simulation tool used is the open-source code FAST (Fatigue, Aerodynamics, Structures and Turbulence) from National Renewable Energy Laboratory (NREL). v8.16.00a-bjj is utilised in this paper. FAST is a very widely used, fully coupled aero-hydro-elastic-servo wind turbine analysis tool. FAST consists of five modules, namely, AeroDyn [20], ElastoDyn [21], HydroDyn [22], ServoDyn, and MoorDyn [23]. As the names of the modules suggest, they provide the modelling of aerodynamics, structural dynamics, hydrodynamics, control dynamics and mooring dynamics, respectively. These modules work in combination to provide the modelling and simulation of the 10 MW OO-star FWT. FAST is a time-domain simulation tool that uses time-domain stochastic wind and waves as inputs. Some examples of FAST being used are [[24], [25], [26]]. FAST has also been benchmarked and compared to other codes in several global code comparison studies where many researchers participated. These examples include OC3: Offshore Code Comparison Collaboration [27] and OC4: IEA (International Energy Agency) Task Wind 30 [28] and the comparison study performed in the Netherlands [29].

### 3.2 Extreme value prediction

The largest maxima extracted from a group of individual maxima, i.e., the extreme value, in a stochastic process *X(t)* within a time span (0,*T*) can be presented as in Eq. (1).

(1) $$X_{e}=\max\left\{X_{m1},X_{m2},X_{m3},\ldots .,X_{mn}\right\},i=1,\ldots ,n$$

where *X*_*e*_ is the largest maximum value and *X*_*mi*_ is the individual maxima. From Eq. (1), one can than define that a common function *F*_*Xm*_*(x)* where within it has independent and identically distributed maxima values. Following this, Eq. (2) can written:

(2) $$\left(x\right)=Prob\left\{X_{e}\leq x\right\}={\left[F_{Xm}\left(x\right)\right]}^{n},i=1,\ldots ,n$$

There are several methods available to model an extreme value distribution. Some examples where extreme value methods have been applied to wind turbines are Cheng et al. [30], which studied a floating vertical axis wind turbine, and Xu et al. [31], where the effects of wave nonlinearity on extreme loads on a semi-submersible FOWT was studied. The two methods used in this paper are the ACER method (Section 3.3) and the Gumbel method (Section 3.4).

### 3.3 ACER (average conditional exceedance rate)

This paper uses the ACER method to estimate extreme structural responses. The method was proposed by Naess and Gaidai [32], and it is derived from a discretely sampled response process. The cascade of conditional approximation is the basis for calculating the exceedance probability for extreme value estimation.

To accurately model the distribution function of the extreme value is the primary purpose of the ACER method; the extreme value function is defined as $M_{N}=\max\left\{X_{j};\;j=1,\cdots ,N\right\}$. The probability of the occurrence of the extreme value η is $P_{\eta }=Prob\left(M_{N}\leq \eta \right)$ and can be written as in Eq. (3):

(3) $$P_{\eta }=Prob\left(M_{N}\leq \eta \right)=Prob\left(X_{1}\leq \eta ,\cdots ,X_{N}\leq \eta \right)$$

To solve this equation efficiently, a cascade of conditional approximation $P_{k}\left(\eta \right)$ is used, where $P_{k}\left(\eta \right)$ tends to close to $P_{\eta }$ as k increases. For $N^{\prime \prime }1$ and k=1,2,⋯, $P_{k}\left(\eta \right)$ is represented as:

(4) $$P_{k}\left(\eta \right)\approx \exp\left(-\sum_{j=k}^{N}\alpha_{kj}\left(\eta \right)\right)$$

where $\alpha_{kj}\left(\eta \right)=Prob\left(X_{1}>\;\eta |{X_{j-1}}^{\prime \prime }\eta ,\cdots ,X_{j-k+1}\leq \eta \right)$, and it represents the exceedance probability conditional on k−1 previous non-exceedances.

Eq. (4) will be calculated based on the ACER, which is defined as in Eq. (5):

(5) $$\varepsilon_{k}\left(\eta \right)=\frac{1}{N-k+1}\sum_{j=k}^{N}\alpha_{kj}\left(\eta \right),k=1,2,\cdots$$

For k≥ 2, ${\tilde{\varepsilon }}_{k}\left(\eta \right)$ is used instead of $\varepsilon_{k}\left(\eta \right)$ because it is easier to use for long-term or nonstationary statistics, and it is defined as given in Eq. (6):

(6) $${\tilde{\varepsilon }}_{k}\left(\eta \right)=\lim_{N\to \infty }\frac{\sum_{j=k}^{N}a_{kj}\left(\eta \right)}{N-k+1}$$

where $a_{kj}\left(\eta \right)$ is the realised values for the observed time series, and $\lim_{N\to \infty }\frac{{\tilde{\varepsilon }}_{k}\left(\eta \right)}{\varepsilon_{k}\left(\eta \right)}=1\text{.}$

The sample estimate of the ACER can be denoted as given by Eq. (7):

(7) $${\hat{\varepsilon }}_{k}\left(\eta \right)=\frac{1}{R}\sum_{r=1}^{R}{{\hat{\varepsilon }}_{k}}^{\left(r\right)}\left(\eta \right)$$

where *R* is the number of samples, and

(8) $${{\hat{\varepsilon }}_{k}}^{\left(r\right)}\left(\eta \right)=\frac{\sum_{j=k}^{N}{a_{kj}}^{\left(r\right)}\left(\eta \right)}{N-k+1}$$

where *r* denotes the realisation number in Eq. (8).

Equations (7), (8) apply for both stationary and nonstationary time series. When the realisations are sufficiently numerous and assumed to be independent, then the 95% confidence interval (CI) can be estimated as given by Eq. (9):

(9) CI(η)=εˆk(η)±1.96sˆk(η)R

where ${\hat{s}}_{k}\left(\eta \right)$ refers to the standard deviation of samples, which is then estimated by using Eq. (10):

(10) $${{\hat{s}}_{k}\left(\eta \right)}^{2}=\frac{1}{R-1}\sum_{r=1}^{R}{\left({{\hat{\varepsilon }}_{k}}^{\left(r\right)}\left(\eta \right)-{\hat{\varepsilon }}_{k}\left(\eta \right)\right)}^{2}$$

The above equations for estimating average exceedance rate are based on direct numerical simulations. In contrast, an extrapolation technique can reduce computational time.

The mean exceedance rate in the tail is assumed to behaves similarly to $\exp\left\{{-a\left(\eta -b\right)}^{c}\right\}$ ($\eta \geq \eta_{0}\geq b$). The parameters *a*, *b* and *c* are suitable constants to be found via fitting. Eq. (11) then writes the ACER function:

(11) $$\varepsilon_{k}\left(\eta \right)\approx q_{k}\left(\eta \right)\exp\left\{{-a_{k}\left(\eta -b_{k}\right)}^{c_{k}}\right\},\eta \geq \eta_{0}$$

where the function $q_{k}\left(\eta \right)$ varies slowly compared to the exponential function $\exp\left\{{-a_{k}\left(\eta -b_{k}\right)}^{c_{k}}\right\}$ in the tail region, thus it can be replaced by a constant $\eta_{0}$ when a suitable tail marker is chosen.

Finally, the constants *a*, *b*, *c* and *q* can be estimated using the Levenberg-Marquardt least-squares optimisation method. Following this, the ACER method can obtain the probability of the occurrence of the extreme value. It is mentioned that Naess et al. [33] and Chai et al. [34] have shown that the ACER method is very computationally efficient while being satisfactory accurate. The ACER method is described in detail in Ref. [35].

### 3.4 Gumbel fitting method

The extreme value distribution Eq. (2) converges in generally for large sample sizes (*n*) to the Gumbel, Fréchet or Weibull distribution, also known as the Type I, II and III extreme value distributions, respectively. They are a family of cumulative probability distribution that combines the generalised extreme value (GEV) distribution as given in Eq. (12).

(12) $$F_{X_{e}}\left(x\right)=\exp\left(-{\left(1+\gamma \left(\frac{x-\mu }{\beta }\right)\right)}^{-\frac{1}{\gamma }}\right)$$

where *β* is the scale parameter, γ is the shape parameter, and μ is the location parameter. The limiting of γ → 0 allows the approximation to fit the Gumbel distribution, commonly used as a recommendation when modelling marine structures [36].

(13) $$F_{X_{e}}\left(x\right)=\exp\left(-\exp\left(-\frac{x-\mu }{\beta }\right)\right)$$

Eq. (13) can be rewritten by using a logarithm on the equation, allowing it to become a linear function as presented in Eq. (14).

(14) $$-\ln\left(\ln\left(F_{X_{e}}\left(x\right)\right)\right)=\frac{x}{\beta }-\frac{\mu }{\beta }$$

*β* and *μ* can be approximated from the original data using the least-square fitting method directly from the cumulative distribution probability [37].

### 3.5 Environmental conditions and load cases

**Fig. 4:**
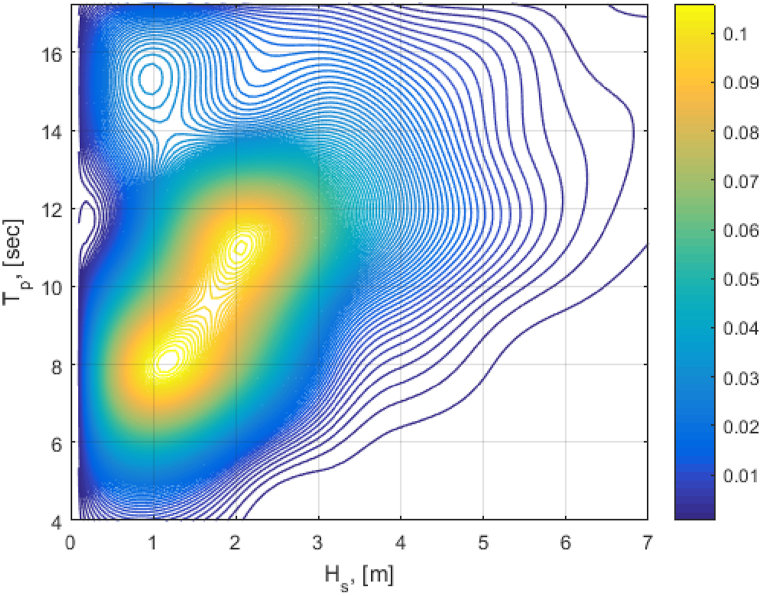
Scattered diagram: In situ values of *H*_s_ and *T*_p_ used to assign probabilities for the individual sea states.

Environmental conditions based on hindcast data measured at site 14 in Lin et al. [38] from the North Sea from 2001 to 2020 are used. The long-term joint wind and wave distribution are described below in Eq. (15):

(15) $$f_{U_{10,}H_{s,}T_{p}}\left(u,h,t\right)=f_{U_{10}}\left(u\right)∙f_{H_{s}{ǀU}_{10}}\left(hǀu\right)∙f_{T_{p}{ǀU}_{10,}H_{s}}\left(tǀu,h\right)$$

where U10 is the 1-h mean wind speed located 10 m above the sea level, Tp is the wave spectral peak period, Hs is the significant wave height, and the marginal distribution of *U*_*10*_ is described by $f_{U_{10}}\left(u\right)$ *,* $f_{H_{s}{ǀU}_{10}}\left(hǀu\right)$ and $f_{T_{p}{ǀU}_{10,}H_{s}}\left(tǀu,h\right)$, the conditional distribution of *H*_*s*_ for given *U*_*10*_ and the conditional distribution of *T*_*p*_ for given *U*_*10*_ and *H*_*s*_ (see Fig. 3). Fig. 4 shows a scattered diagram for the in situ values of *H*_*s*_ and *T*_*p*_ that are used to assign probabilities for the individual sea states.

To replicate a highly probabilistic normal operational condition experienced by the turbine, three closely related load cases were selected and presented in Table 4. These load cases presented the cut-in, rated and cut-out zones of the FOWT. Each load case has its corresponding Hs and Tp in accordance with the joint distribution presented in Eq. (15). The irregular waves and turbulent wind are aligned in direction. Wind turbine Class C is used with normal turbulence and normal wind profiles. The wind power-law formulation described in Eq. (16) is used to model the wind speed profile.

(16) $$U_{w}\left(z\right)={U_{hub}\;\left(\frac{Z}{Z_{hub}}\right)}^{\alpha }$$

where *U*_*w*_*(z)* is the mean wind speed taken from height z above the still water level, *u*_*hub*_ is the mean wind speed w. r.t hub height, *z*_*hub*_ is the hub height w. r.t the still water level (119 m for the selected 10-MW FOWT). *α* (power-law exponent) is equal to 0.14. These recommendations are from IEC 61400-3-2, see Ref. [39], used for offshore locations.

**Table 4:** Load cases for numerical simulations.

Load cases	$U_{w}$ (m/s)	$T_{I}$	$H_{s}$ (m)	$T_{p}$ (s)	Samples	Simulation length (s)
LC1	8	0.1740	1.9	9.7	20	4000
LC2	12	0.1460	2.5	10.1	20	4000
LC3	16	0.1320	3.2	10.7	20	4000

The 3-D wind turbulent fields generated using Turbsim is derived from Kaimal's turbulence model [40]. At the same time, the JONSWAP (Joint North Sea Wave Project) spectrum allowed the modelling of the time-varying irregular waves with the respective Hs and Tp values.

Every simulation was conducted for a period of 4000s. The initial 400s of these simulations were disregarded to account for the transient effect often present during a turbine's start-up. Consequently, only 3600s of data is used to analyse the extreme value. Accordingly, each environmental condition had sea states with 20 random wind and wave conditions samples.

## 4 Response variables

The following response variables are considered

• RootMyb1: blade 1 root flapwise bending moment
• LSSTipMys: main shaft tip up-down bending moment
• TwrBsMyt: tower bottom fore-aft bending moment

**Fig. 5:**
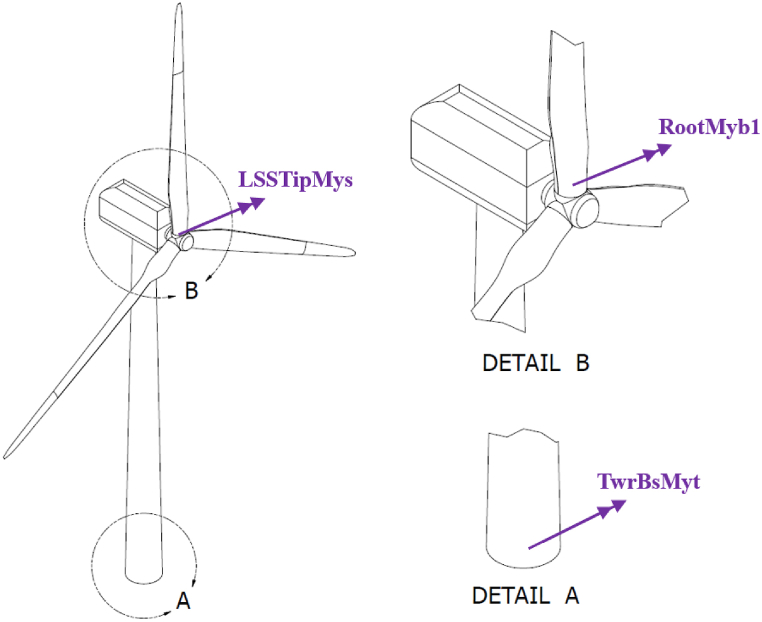
Location of points where bending moments are measured. Detail A: Close up at tower bottom. Detail B: Close up at rotor.

The locations where these response variables are measured are presented in Fig. 5.

## 5 Results and discussions

### 5.1 Time-domain responses, PSD, and maximum values

**Fig. 6:**
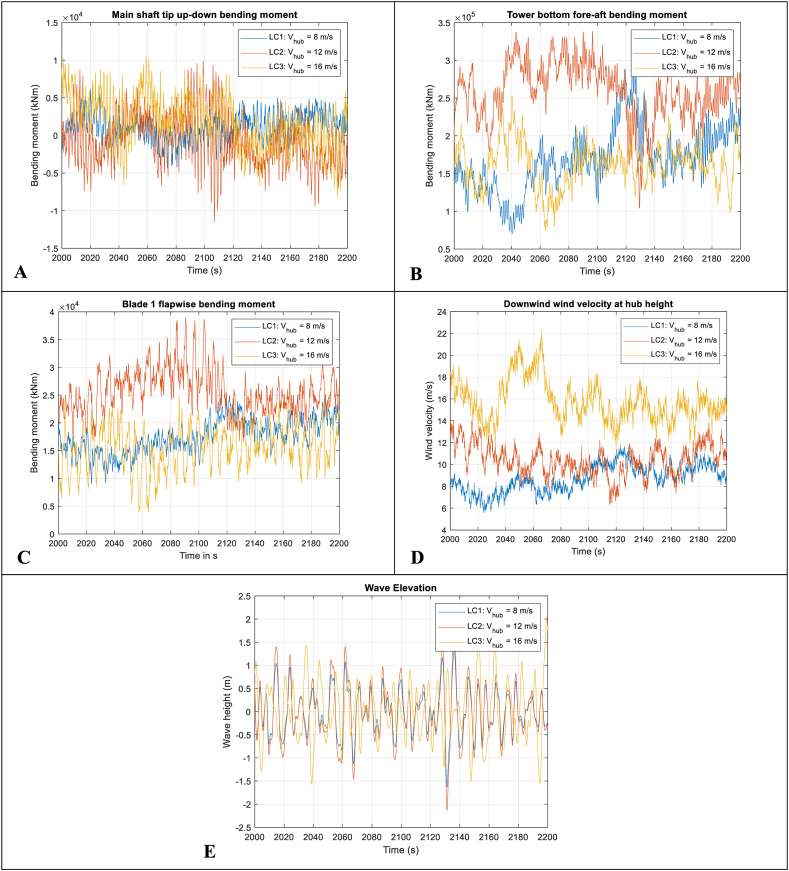
Example time domain results. A-Top-left: Main shaft tip up-down bending moment (LSSTipMys); B-Top-right: Tower bottom fore-aft bending moment (TwrBsMyt); C-Centre-left: Blade 1 root flapwise bending moment (RootMyb); D-Centre-right: Downwind wind velocity at hub height; E-Bottom: Wave elevation.

**Fig. 7:**
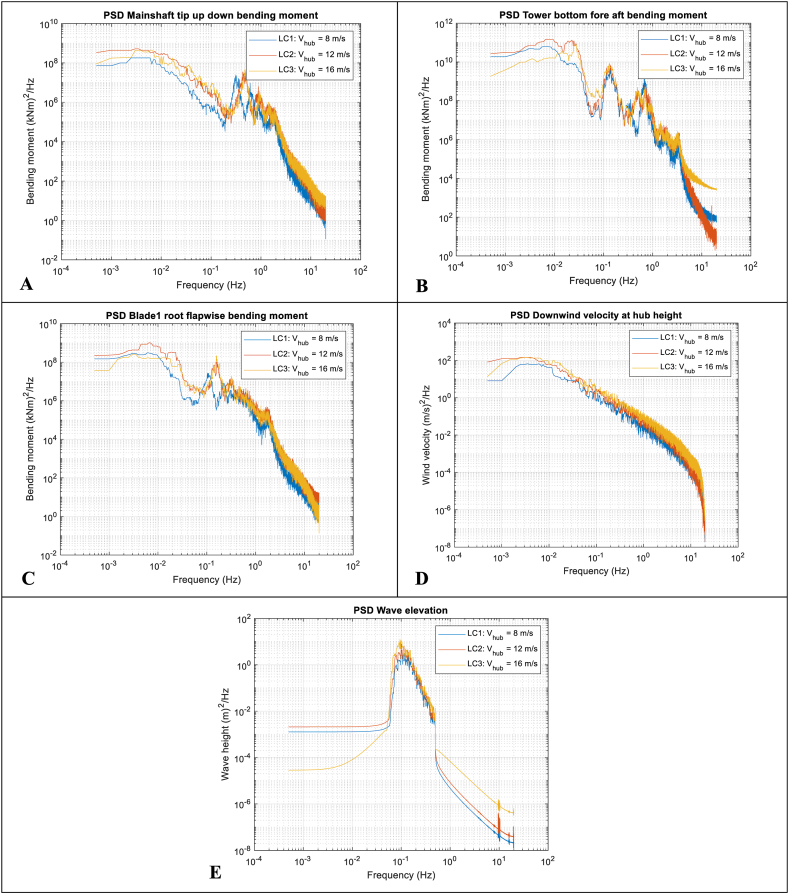
Power spectral distributions. A-Top-left: Main shaft tip up-down bending moment (LSSTipMys); B-Top-right: Tower bottom fore-aft bending moment (TwrBsMyt); C-Centre-left: Blade 1 root flapwise bending moment (RootMyb); D-Centre-right: Downwind wind velocity at hub height; Bottom: E-Wave elevation.

**Fig. 8:**
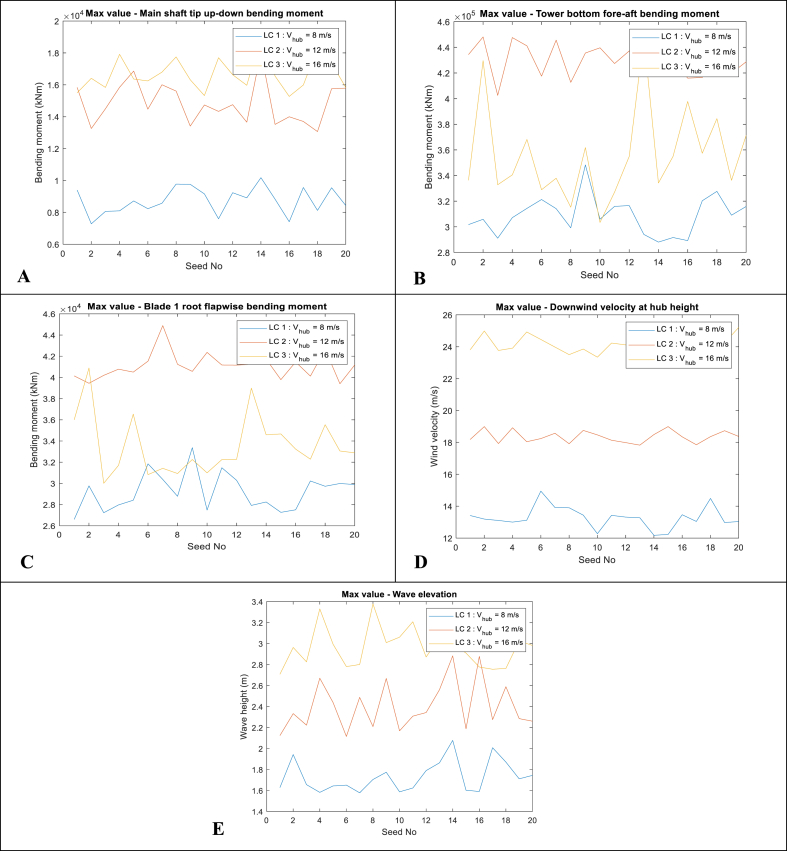
Maximum value in each realisation. A-Top-left: Main shaft tip up-down bending moment (LSSTipMys); B-Top-right: Tower bottom fore-aft bending moment (TwrBsMyt); C-Centre-left: Blade 1 root flapwise bending moment (RootMyb); D-Centre-right: Downwind wind velocity at hub height; E-Bottom: Wave elevation.

The time-domain responses for one portion of a realisation, the power spectral distributions (PSDs) for a full realisation and the maximum values of each realisation are presented in Figs. 6, 7, and 8, respectively. These load case results, i.e., LC1, LC2 and LC3, are taken from one of the 20 realisations calculated. The wind and wave elevation time series and PSDs are also plotted for reference.

### 5.2 Extreme load responses using ACER and Gumbel methods

**Fig. 9:**
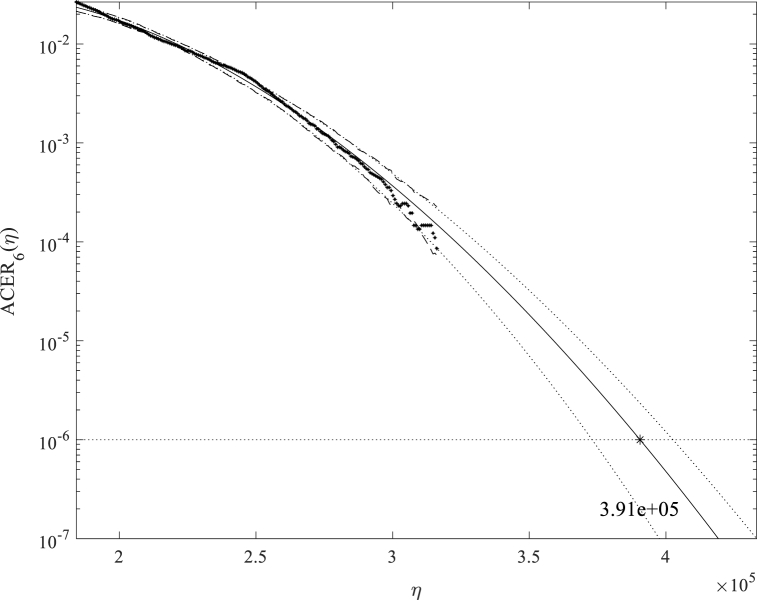
Example plot of ACER extrapolation, TwrBsMyt, LC1 – V_hub_ = 8 m/s, Realisation #1.

**Fig. 10:**
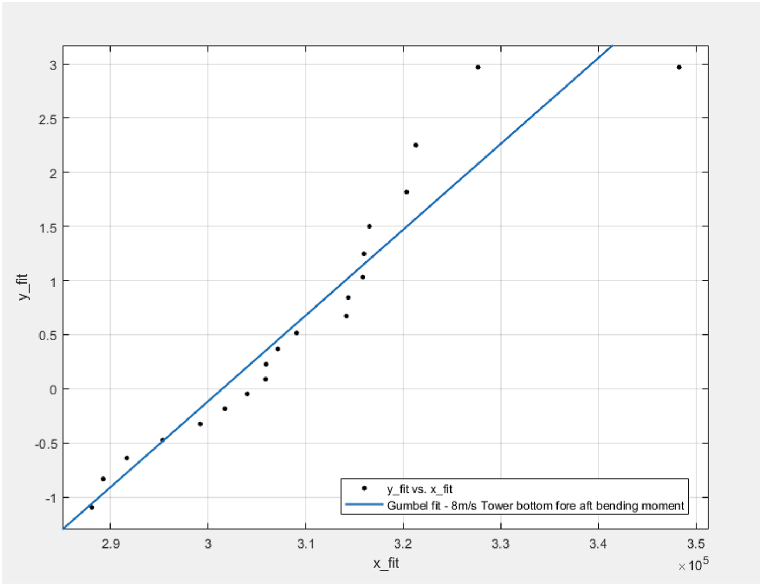
Example plot of Gumbel fitting, TwrBsMyt, LC1 – V_hub_ = 8 m/s, Realisation #1; x_fit = x, y_fit = −ln (−ln (F(x)).

This section presents the extreme load responses using the ACER and Gumbel methods for the three operating conditions (LC1 – LC3) presented in Table 4. *k* = 6 is used. For illustration, Fig. 9, Fig. 10 present example plots of the ACER extrapolation and Gumbel fitting, respectively.

As illustrated by the significantly smaller confidence intervals, the ACER method can lead to more accurate results as it does not assume a distribution. No extreme distribution is assumed in the ACER model. Instead, it follows the exact shape of the data points as presented in Fig. 9. On the other hand, from Fig. 10, the upper-end tail is not well-fitted when the Gumbel distribution is used. The data points curve towards the left for increasing response values and are above the Gumbel line. This means the Gumbel distribution will tend to overpredict the extreme value responses for most responses, particularly for the above-rated cases. This example shows the advantages of the ACER method.

**Fig. 11:**
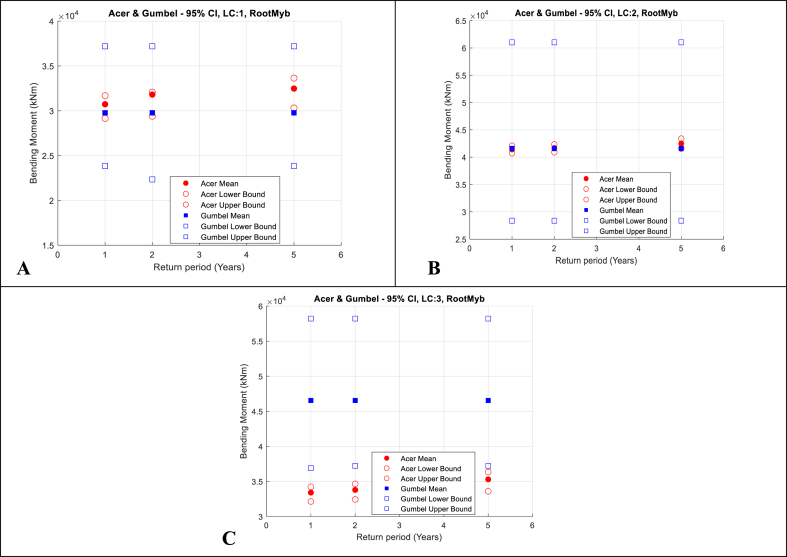
Blade 1 root flapwise bending moment. ACER and Gumbel with 95% CI; A-Top-left: LC1, V_hub_ = 8 m/s; B-Top-right: LC2, V_hub_ = 12 m/s; C-Bottom: LC3, V_hub_ = 16 m/s.

**Fig. 12:**
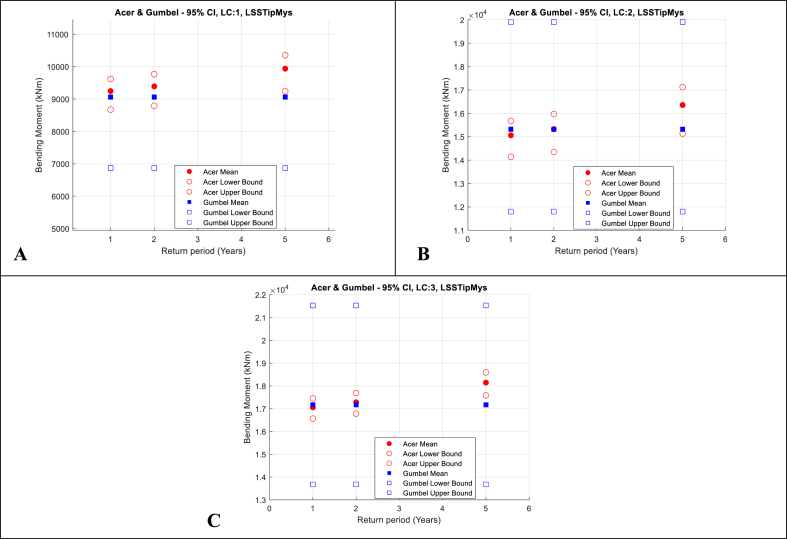
Main shaft tip up-down bending moment. ACER and Gumbel with 95% CI; A-Top-left: LC1, V_hub_ = 8 m/s; B-Top-right: LC2, V_hub_ = 12 m/s; C-Bottom: LC3, V_hub_ = 16 m/s.

**Fig. 13:**
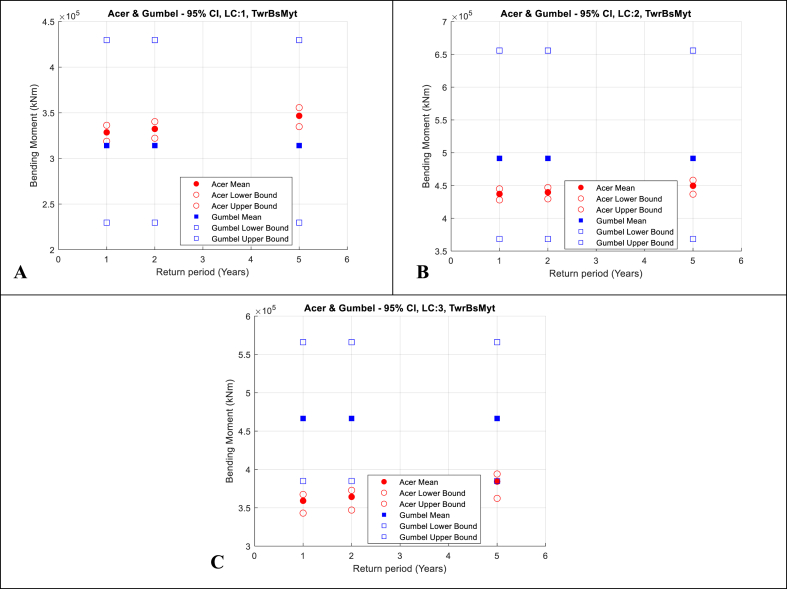
Tower bottom fore-aft bending moment. ACER and Gumbel with 95% CI; A-Top-left: LC1, V_hub_ = 8 m/s; B-Top-right: LC2, V_hub_ = 12 m/s; C-Bottom: LC3, V_hub_ = 16 m/s.

The extreme load responses and the 95% CIs from both ACER and Gumbel methods are then plotted in Fig. 11, Fig. 12, Fig. 13 for RootMyb, LSSTipMys and TwrBsMyt, respectively. Table 6 and Table 7 of the Appendix present numerical values of these results for extreme values calculated by the ACER and Gumbel methods, respectively.

The following observations are made:

• The 1, 2 and 5-year extreme values are generally 1.1–1.3 times larger than the maximums of single 1-h realisations. The relatively large range of values (about 20%) indicates the importance of using extrapolation methods that are accurate in predicting extreme values that can be used to define appropriate design values that can be utilised in deterministic engineering design.
• The 95% CIs of the results calculated using the ACER method are significantly smaller than those of the Gumbel method. This highlights the benefits of the ACER in not assuming a distribution in the extrapolation of extreme values.
• The 95% CIs of the results calculated using the Gumbel method are larger. This indicates that the Gumbel distribution does not fit the extreme value responses well.
• Further, the 1, 2, and 5-year extreme values calculated using the Gumbel method are relatively similar. This is because the upper tail end is not accurately fitted, and the fitted Gumbel probability density distribution slope is too steep at the upper tail end. This leads to minimal changes in the response values for a unit change in probability.

### 5.3 Choice of *k* value in ACER method

**Fig. 14:**
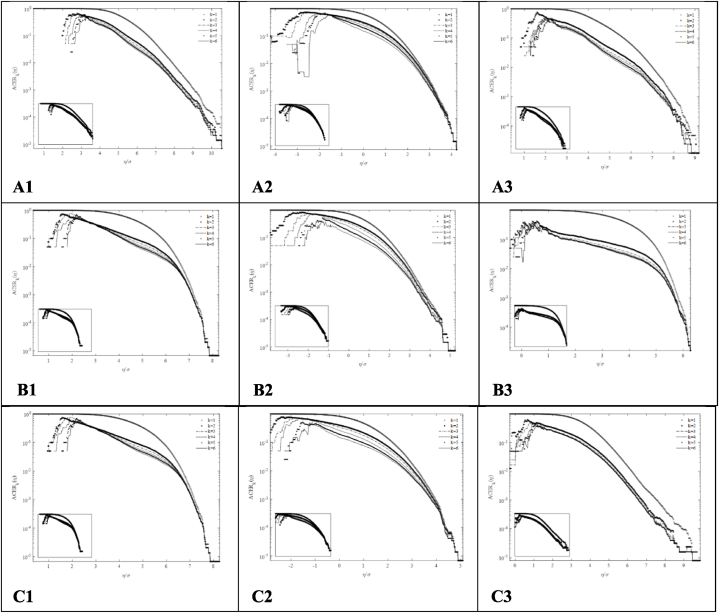
ACER functions for various k values. A-Top: LC1, V_hub_ = 8 m/s; B-Centre: LC2, V_hub_ = 12 m/s; *C*-Bottom: LC3, V_hub_ = 16 m/s; 1-Left: Blade 1 root flapwise bending moment (RootMyb); 2-Centre: Mainshaft tip up-down bending moment (LSSTipMys); 3-Right: Tower bottom fore-aft bending moment (TwrBsMyt).

It is recommended to perform sensitivity analyses of the *k* values when studying new responses [32]. Therefore, the choice of *k* value is investigated in this section for a *q* value of 10^−6^. The results for *k* = 2, 4 and 6 are presented in Table 5. The ACER function plots for *k* = 1 to 6 are presented in Fig. 14.

**Table 5:** Extreme values calculated from the ACER method considering different values of k.

Load Case	*q* value	10^–6^		
	*k* value	2	4	6
LC1, V_hub_ = 8 m/s	RootMyb (kNm)	36,103	36,441	37,018
	LSSTipMys (kNm)	11,509	11,580	11,592
	TwrBsMyt (kNm)	380,076	368,822	390,514
LC2, V_hub_ = 12 m/s	RootMyb (kNm)	44,536	44,626	44,951
	LSSTipMys (kNm)	19,511	19,228	19,607
	TwrBsMyt (kNm)	476,606	479,938	480,159
LC3, V_hub_ = 16 m/s	RootMyb (kNm)	38,980	40,385	40,214
	LSSTipMys (kNm)	20,747	20,803	20,670
	TwrBsMyt (kNm)	450,802	447,620	450,217

The extreme values calculated do not vary significantly with the value of *k* used. A *k* value of 1 was found to lead to incorrect results. The extreme values estimated also increase for increasing values of *k* used. It was observed that the responses converged for *k* > 2. Therefore, it was decided to use *k* = 6 for the analyses in this paper.

## 6 Conclusions

The extreme responses for the 10 MW OO-star FOWT using ACER and Gumbel methods were studied using numerical results from FAST simulations. The following conclusions are made:

• The 1, 2 and 5-year responses of the FOWT were generally 1.1–1.3 times larger than the maximums of single 1-h realisations. This reinforces the importance of using extrapolation methods to determine extreme loads to be used as ULS loads.
• The ACER method is more accurate as its results have a smaller 95% CI than the Gumbel results.
• The 1, 2 and 5-year responses predicted by the Gumbel method are quite similar. This is due to the poorly fitted upper tail by the Gumbel method. In contrast, the ACER does not assume any distributions and therefore does not have the same poor fit issue at the tail end.
• The better performance of the ACER method is because, in contrast to Gumbel, it does not assume that the extreme responses follow a designated probability distribution.

It was found that *k* = 1 would lead to incorrect results and cannot be used, but otherwise, the choice of the *k* values does not affect the ACER results. When new responses are studied, it is also recommended to perform sensitivity studies on the k values.

Although this paper only focuses on a particular FWT concept, it is noted that, in general, it is of interest to investigate the extreme responses and dynamic behaviour of a wind turbine facility supported on different substructures, such as Monopile, Spar, and TLP.

## Author contribution statement

Yihan Xing; Shuaishuai Wang, PhD: Conceived and designed the experiments; Performed the experiments; Analyzed and interpreted the data; Contributed reagents, materials, analysis tools or data; Wrote the paper.

Anuraj Karuvathil; Rajiv Balakrishna: Analyzed and interpreted the data; Wrote the paper.

Oleg Gaidai: Contributed reagents, materials, analysis tools or data; Wrote the paper.

## Funding statement

This research did not receive any specific grant from funding agencies in the public, commercial, or not-for-profit sectors.

## Data availability statement

Data included in article/supp. material/referenced in article.

## Declaration of interest's statement

The authors declare no conflict of interest.

## Figures and Tables

**Table 6:** Extreme value responses using ACER method for various return periods; 95% confidence interval in paratheses.

Load Case	Return period	1 yr	2 yr	5 yr
	Exceedance probability, q	7.19 × 10^−5^	5.71 × 10^−5^	2.28 × 10^−5^
LC1, V_hub_ = 8 m/s	RootMyb (kNm)	30,726 (29,149, 31,682)	31,084 (29,393, 32,082)	32,479 (30,315, 33,645)
	LSSTipMys (kNm)	9245 (8673, 9616)	9390 (8792, 9768)	9937 (9241, 10,351)
	TwrBsMyt (kNm)	328,555 (318,797, 336,312)	332,314 (322,130, 340,328)	346,662 (334,792, 355,663)
LC2, V_hub_ = 12 m/s	RootMyb (kNm)	41,445 (40,752, 42,073)	41,662 (40,934, 42,338)	42,485 (41,615, 43,372)
	LSSTipMys (kNm)	15,061 (14,140, 15,677)	15,327 (14,349, 15,973)	16,357 (15,136, 17,122)
	TwrBsMyt (kNm)	437,049 (428,032, 444,853)	439,643 (429,627, 446,865)	449,561 (436,751, 457,982)
LC3, V_hub_ = 16 m/s	RootMyb (kNm)	33,406 (32,141, 34,231)	33,790 (32,440, 34,657)	35,293 (33,587, 36,335)
	LSSTipMys (kNm)	17,054 (16,567, 17,449)	17,282 (16,780, 17,688)	18,144 (17,584, 18,591)
	TwrBsMyt (kNm)	359,140 (343,069, 367,411)	364,244 (347,041, 372,826)	384,245 (362,296, 394,070)

**Table 7:** Extreme value responses using Gumbel method for various return periods; 95% confidence interval in paratheses.

Load Case	Return period	1 yr	2 yr	5 yr
	Exceedance probablity, q	7.19 × 10^−5^	5.71 × 10^−5^	2.28 × 10^−5^
LC1, V_hub_ = 8 m/s	RootMyb (kNm)	29,772 (23,844, 37,194)	29,772 (22,344, 37,194)	29,772 (23,844, 37,195)
	LSSTipMys (kNm)	9061 (6870, 11,953)	9061 (6870, 11,953)	9061 (6870, 11,953)
	TwrBsMyt (kNm)	314,083 (229,636, 429,680)	314,083 (229,636, 429,680)	314,084 (229,636, 429,681)
LC2, V_hub_ = 12 m/s	RootMyb (kNm)	41,593 (28,380, 61,026)	41,593 (28,380, 61,026)	41,593 (28,380, 61,026)
	LSSTipMys (kNm)	15,321 (11,801, 19,903)	15,321 (11,801, 19,904)	15,321 (11,801, 19,904)
	TwrBsMyt (kNm)	491,364 (368,347, 655,777)	491,364 (368,347, 655,778)	491,365 (368,348, 655,779)
LC3, V_hub_ = 16 m/s	RootMyb (kNm)	46,542 (36,896, 58,215)	46,542 (37,218, 58,215)	46,542 (37,218, 58,215)
	LSSTipMys (kNm)	17,168 (13,676, 21,530)	17,168 (13,676, 21,530)	17,168 (13,676, 21,530)
	TwrBsMyt (kNm)	466,508 (384,880, 566,002)	466,510 (384,880, 566,003)	466,510 (384,882, 566,004)
